# Distinct metabolomic signatures of SGLT2 inhibitor vs. sulfonylurea in a paired human liver biopsy study

**DOI:** 10.1016/j.bbrep.2026.102784

**Published:** 2026-09-14

**Authors:** Yumie Takeshita, Hein Ko Oo, Yujiro Nakano, Ayano Ueno, Maki Oishi, Hiromasa Tsujiguchi, Tomoyoshi Soga, Taro Yamashita, Masao Honda, Toshinari Takamura

**Affiliations:** a Department of Endocrinology and Metabolism, Kanazawa University Graduate School of Medical Sciences, 13-1 Takara-machi, Kanazawa, Ishikawa, 920-8640, Japan; b Department of Gastroenterology, Kanazawa University Graduate School of Medical Sciences, 13-1 Takara-machi, Kanazawa, Ishikawa, 920-8640, Japan; c Department of Environmental and Preventive Medicine, Faculty of Medicine, Institute of Medical, Pharmaceutical and Health Sciences, Kanazawa University, 13-1 Takara-machi, Kanazawa, 920-0934, Japan; d Institute for Advanced Biosciences, Keio University, 246-2 Mizukami, Kakuganji, Tsuruoka, 997-0052, Japan; e Human Biology-Microbiome-Quantum Research Center (WPI-Bio2Q), Keio University, Tokyo, 108-8345, Japan

## Abstract

This study aimed to elucidate how sodium-glucose cotransporter 2 (SGLT2) inhibitors and sulfonylureas differentially modulate hepatic metabolism in persons with metabolic dysfunction-associated steatotic liver disease (MASLD) and type 2 diabetes (T2D).

In this 48-week randomized, open-label, parallel-group trial, Japanese participants with type 2 diabetes and biopsy-confirmed MASLD received either tofogliflozin or glimepiride. Paired liver biopsy samples and serum were collected at baseline and after 48 weeks. We performed a post hoc metabolomic analysis to assess hepatic and systemic metabolic shifts and their correlations with histological and clinical parameters.

Tofogliflozin treatment significantly increased hepatic glucose 1-phosphate and acetyl-CoA levels, reflecting a metabolic shift toward ketogenesis and gluconeogenesis. Regarding TCA cycle intermediates, tofogliflozin elevated serum citrate but reduced serum succinate; notably, the reduction in succinate correlated with reduction in liver enzyme levels. Furthermore, higher baseline serum succinate predicted superior histological improvements (ballooning and inflammation) in the tofogliflozin group. In contrast, glimepiride specifically reduced hepatic branched-chain amino acids (BCAAs), which was associated with decreased steatosis. Tofogliflozin also elevated serum urea cycle intermediates, suggesting a distinct “aestivation-like” response to hypovolemia.

The metabolic signature of SGLT2 inhibitors in the human liver is characterized by starvation-induced glucose production and hypovolemia-induced aestivation-like response. Serum succinate emerged as a potential biomarker associated with MASLD severity and treatment-related improvements under SGLT2 inhibition.

## Highlights

• SGLT2 inhibitors shift toward starvation-induced endogenous glucose production, distinct from the anabolic profile of glimepiride.
• SGLT2 inhibition triggered an aestivation-like response via modulation of urea cycle intermediates.
• Serum succinate may be a non-invasive biomarker reflecting improvement in liver pathology.

## Graphical abstract

**Figure:**
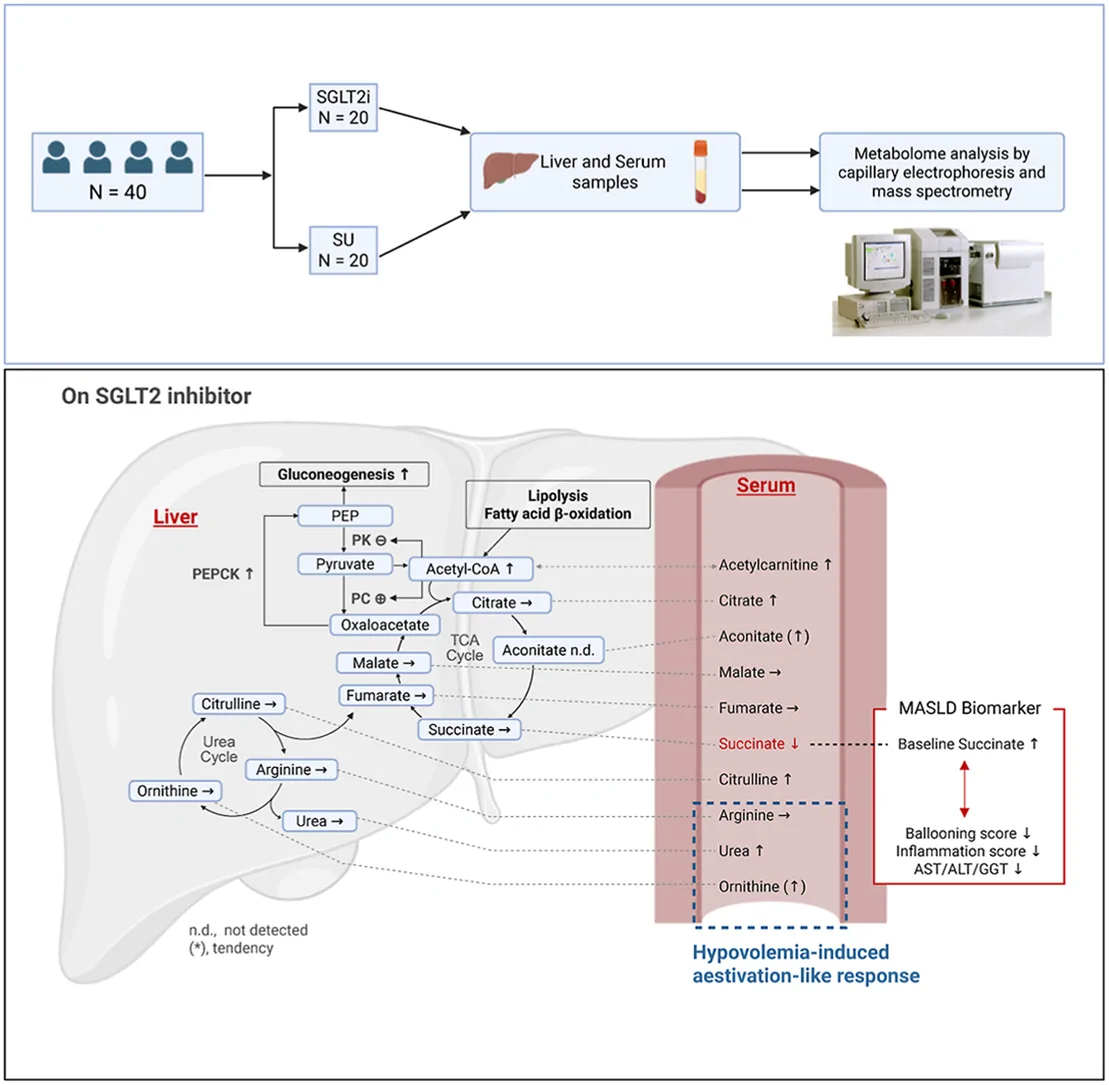

## 1 Introduction

The liver plays a central role in macronutrient metabolism, and its perturbation contributes to the development of type 2 diabetes (T2D) and its clinical manifestations. Metabolic dysfunction-associated steatotic liver disease (MASLD) encompassing a spectrum from simple steatosis to metabolic dysfunction-associated steatohepatitis (MASH), is a hallmark liver phenotype of metabolic disorders such as T2D and obesity [1]. MASLD and T2D share reciprocal epidemiological and pathophysiological links; hyperglycemia is a critical driver of liver fibrosis [2,3], cirrhosis, hepatocellular carcinoma, and poor prognosis in MASH [[4], [5], [6]].

Sodium-glucose cotransporter-2 (SGLT2) inhibitors exert beneficial hemodynamic and metabolic effects, reducing hemoglobin A1c (HbA1c), body weight, and serum liver enzymes in MASLD [7,8]. While large-scale clinical trials have established their cardioprotective and renoprotective benefits in T2D, their definitive efficacy in MASLD remains under investigation. We previously demonstrated that the SGLT2 inhibitor tofogliflozin significantly improved liver pathology compared to the sulfonylurea glimepiride, despite achieving similar glycemic control [7]. This improvement was accompanied by dynamic shifts in hepatic gene expression related to energy metabolism, inflammation, and fibrosis [7]. These findings suggest that SGLT2 inhibitors may uniquely modulate hepatic metabolism, though the underlying metabolic signatures remain poorly understood.

Metabolomic analysis of serum and liver tissues provides deep insights into MASLD pathophysiology. In diabetic mice, dapagliflozin has been shown to increase hepatic and plasma levels of specific amino acids, such as valine and leucine, in correlation with its therapeutic effects [9]. However, although several studies have characterized the serum and plasma metabolomes, the impact of SGLT2 inhibitors on the hepatic metabolome remains unexplored in humans.

In this exploratory post hoc analysis, we characterized the changes in serum and hepatic metabolites among Japanese participants with biopsy-confirmed MASLD and T2D treated with either the SGLT2 inhibitor tofogliflozin or the sulfonylurea glimepiride. These two agents provide a unique contrast: both achieve comparable glucose reduction, yet they exert opposing effects on circulating insulin levels (reduction vs. elevation).

## 2 Results

### 2.1 Participant characteristics

**Fig. 1:**
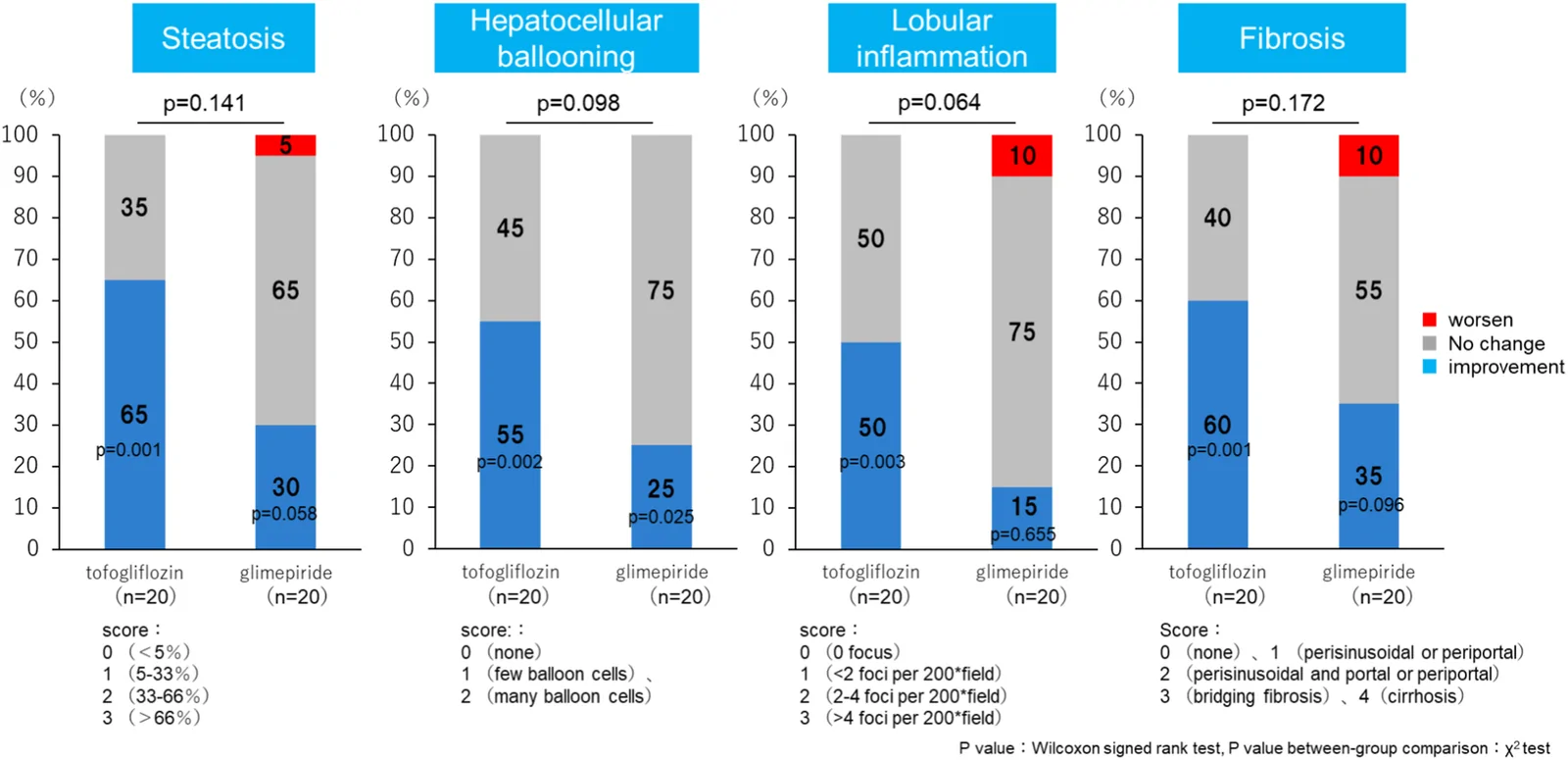
Patients with changes in hepatic histological scores.

Forty participants were randomly assigned to receive tofogliflozin once daily at a dose of 20 mg (20 participants) or glimepiride once daily at a final mean dose of 0.8 mg (20 participants). The mean age, NAS, HbA1c level, and weight were 53.9 years, 4.45, 8.2, and 82.0 kg, respectively. Altogether, 18 (45.0%), 11 (27.5%), and 5 (12.5%) participants from a previous paper had stages F1, F2, and F3 fibrosis, respectively [7]. The reductions in glycemic parameters, including fasting plasma glucose and HbA1c levels, were similar. In a previous study [7], participants receiving tofogliflozin showed significant histological improvements from baseline to 48 weeks in all variables (ratios of the participants with improvement in steatosis, hepatocellular ballooning, lobular inflammation, and fibrosis were 65%, 55%, 50%, and 60%, respectively; Fig. 1). On the other hand, participants receiving glimepiride showed significant histological improvements from baseline to 48 weeks in only hepatocellular ballooning.

In the tofogliflozin group, the levels of serum liver enzymes, including ALT, AST, gamma-glutamyl transferase, weight, and BMI were significantly reduced [7]. The changes in CPR were similar in both groups (0.00 ± 0.23 ng/mL in the tofogloflozin group vs. 0.26 ± 0.15 ng/mL in the glimepiride group) [7]. Urinary CPR levels decreased significantly in the tofogliflozin group (69.5 [39.2–134.0] to 35.4 [19.3–66.2] μg/24h, p = 0.001), with significant differences between the groups (−32.0 [−66.7 to −8.5] μg/24h in the tofogloflozin group vs. 3.8 [−5.7 – 29.7] μg/24h in the glimepiride group, p < 0.001).

### 2.2 Changes in hepatic metabolite signatures

In within-group comparisons, during the intervention, gluconeogenic substrates, such as glucose 1-phosphate and acetyl-CoA, were elevated from baseline in the tofogliflozin group, whereas glucose 6-phosphate and fructose 6-phosphate levels were increased in the liver of the glimepiride group (Table 1). The levels of tricarboxylic acid (TCA) cycle intermediates (malate, citrate, and succinate) did not change in either group.

**Table 1:** Changes in hepatic metabolomics parameters compared between baseline and week 48 by treatment group.

	Tofogliflozin				Glimepiride				
	N	Week 0	Week 48	P*	N	Week 0	Week 48	P*	P**
**Carbohydrate metabolism**									
glucose 1-phosphate (G1P)	16	32.0 (23.0-41.0)	40.0 (35.0-43.8)	0.047	16	43.0 (27.0-55.3)	36.5 (29.5-50.0)	0.814	0.170
glucose 6-phosphate (G6P)	17	240.0 (106.0-360.5)	414.0 (248.5-478.5)	0.149	17	324.0 (138.0-3424.0)	382.0 (339.0-4427.5)	0.039	1.000
fructose 6-phosphate (F6P)	17	50.5 (29.5-78.5)	85.0 (55.0-117.0)	0.088	17	64.0 (25.0-81.0)	77.0 (73.5-102.5)	0.044	0.683
fructose 1,6-bisphosphate (F1,6BP)	17	141.5 (98.8-196.3)	120.0 (67.0-154.5)	0.048	17	159.5 (103.5-219.5)	136.0 (90.0-175.0)	0.090	0.571
lactate	17	6586.0 (4277.5-10398.0)	6295.0 (3957.0-10120.5)	0.586	17	6286.0 (4811.0-14892.5)	5381.0 (3949.5-6636.5)	0.084	0.474
acetyl-CoA	15	22.0 (19.3-27.5)	27.0 (21.8-35.8)	0.028	15	20.0 (17.0-25.0)	26.0 (17.8-32.0)	0.192	0.243
citrate	17	69.0(39.0-207.0)	99.0(44.5-169.0)	0.795	16	76.5(55.0-150.0)	114.0(59.5-165.0)	0.352	0.402
succinate	17	341.0(279.0-476.5)	345.0(285.5-431.0)	0.758	17	351.0(256.5-437.0)	315.0(275.0-424.5)	0.463	0.683
fumarate	17	420.5(195.5-513.8)	491.0(405.5-629.5)	0.332	17	416.0(327.5-597.0)	477.0(408.0-538.0)	0.538	0.973
malate	17	1261.0(882.0-1615.5)	1409.0(1047.0-1654.0)	0.906	17	1189.0(1013.0-1871.0)	1182.0(947.5-1413.5)	0.287	0.496
glycerate	14	383.0(240.5-528.5)	399.0(265.0-944.0)	0.248	15	391.0(258.0-474.0)	303.0(236.0-371.5)	0.078	0.061
**Amino acid metabolism**									
aspartic acid (Asp)	17	534.0(384.0-660.5)	730.0(435.0-1092.0)	0.028	17	865.0(424.0-1440.0)	626.0(406.5-789.5)	0.055	0.007
threonine (Thr)	17	370.0(317.5-747.0)	364.0(312.5-663.0)	0.653	17	467.0(307.5-765.0)	396.0(358.0-471.0)	0.177	0.563
serine (Ser)	17	776.0(637.0-983.0)	852.0(629.5-1124.5)	0.723	17	786.0(559.5-1544.0)	699.0(624.5-939.0)	0.193	0.496
asparagine (Asn)	17	280.0(223.5-474.5)	278.0(255.0-344.0)	0.906	17	297.0(263.5-405.5)	282.0(231.0-30.7.5)	0.084	0.306
glutamine (Gln)	17	3225.0(2906.5-4137)	2963.0(2371.5-3664.5)	0.193	17	2927.0(2375.5-3538.0)	3289.0(2734.5-3476.5)	2.687	0.099
proline (Pro)	17	294.0(202.0-615.5)	241.0(203.0-367.5)	0.850	17	413.0(221.5-902.5)	343.0(249.5-484.5)	0.170	0.563
glycine(Gly)	17	2096.0(1884.0-3424.0)	2129.0(1480.0-2871.0)	0.586	17	2758.0(1570.0-4403.0)	1896.0(1610.0-2146.0)	0.019	0.231
alanine (Ala)	17	1923.0(886.5-2525.5)	1339.0(692.0-2476.0)	0.287	17	2508.0(1305.0-4319.0)	1677.0(980.0-2563.0)	0.246	0.712
valine (Val)	17	229.0(181.0-340.0)	239.0(228.5-306.0)	0.758	17	265.0(217.5-509.5)	219.0(198.5-240.5)	0.026	0.067
isoleucine (Ile)	17	68.0(55.5-119.5)	81.0(64.5-103.0)	0.795	17	93.0(71.0-180.5)	73.0(62.5-80.5)	0.019	0.062
leucine (Leu)	17	141.0(102.5-217.5)	153.0(128.0-213.5)	0.463	17	179.0(137.5-296.0)	138.0(127.5-158.0)	0.044	0.053
lysine (Lys)	17	288.0(236.0-415.0)	285.0(221.0-375.5)	0.463	17	306.0(249.0-616.5)	256.0(230.0-298.5)	0.039	0.658
tyrosine (Tyr)	17	60.0(43.5-114.5)	63.5(50.3-132.5)	0.756	17	76.5(58.7-157.0)	67.0(52.5-81.0)	0.088	0.254
phenylalanine (Phe)	17	45.0(37.0-69.0)	47.0(38.5-73.0)	0.796	17	49.0(37.5-97.0)	45.0(42.5-50.0)	0.118	0.205
histidine (His)	17	501.0(445.5-692.0)	497.0(463.5-653.5)	0.943	17	543.0(439.5-656.0)	534.0(492.0-569.5)	0.795	0.865
**Urea cycle**									
arginine (Arg)	17	32.0(24.0-45.0)	28.0(21.5-39.5)	0.636	17	32.0(24.0-59.0)	27.0(19.0-35.5)	0.033	0.274
urea	17	3801.0(2447.5-5209.5)	4141.0(3375.5-5443.0)	0.177	17	4415.0(3086.0-5330.5)	3473.0(2760.0-4234.5)	0.068	0.012
ornithine	17	221.0(129.0-767.0)	179.0(121.0-305.5)	0.093	17	247.0(145.0-769.5)	173.0(134.5-234.5)	0.046	0.892
citrulline	16	56.0(30.5-96.5)	52.5(49.0-69.8)	0.393	17	79.0(48.0-120.5)	57.0(49.0-80.0)	0.136	0.929
hypoxanthine	15	166.5(107.0-509.8)	226.0(126.0-287.0)	0.570	15	214.0(105.0-397.0)	159.0(111.0-209.0)	0.118	0.539
**Nucleotides metabolism**									
inosine monophosphate (IMP)	13	408.0(266.0-502.0)	552.0(447.0-1086.0)	0.039	15	459.0(344.0-690.0)	484.0(341.5-655.5)	0.865	0.065
adenosine monophosphate (AMP)	17	904.5(460.0-1358.0)	1068.0(776.0-1298.5)	0.586	17	1165.0(777.0-1457.5)	1116.0(939.5-1451.0)	0.723	1.000
adenosine 5′-diphosphate (ADP)	17	1035.0(754.0-1124.0)	957.0(783.0-1162.5)	0.653	17	982.0(583.5-1098.0)	1087.0(1023.5-11302.0)	0.035	0.322
adenosine 5′-triphosphate (ATP)	12	689.5(494.0-987.0)	697.5(489.8-1215.3)	0.937	15	445.0(369.0-717.0)	948.0(583.0-11095.0)	0.023	0.053
ATP/ADP	12	0.60 (0.55-1.01)	0.68(0.54-0.90)	0.530	15	0.45(0.39-0.66)	0.80(0.58-0.92)	0.020	0.010
guanosine 5′-monophosphate (GMP)	15	308.0(255.0-418.0)	352.0(297.5-427.0)	0.670	15	347.0(266.0-405.0)	339.0(263.5-407.0)	0.955	0.775
guanosine 5′-diphosphate (GDP)	15	73.0(65.0-95.0)	82.0(74.0-92.0)	0.423	17	60.0(46.8-85.0)	84.0(61.0-98.5)	0.044	0.342
uridine 5′-diphosphate (UDP)	12	75.5(59.5-93.3)	69.0(44.0-111.0)	0.530	14	49.0(29.0-82.5)	76.0(64.0-104.0)	0.084	0.462
uridine 5′-triphosphate (UTP)	10	65.5(38.8-125.8)	77.0(38.0-116.5)	0.721	13	35.0(19.8-66.8)	84.0(48.3-104.5)	0.087	0.148

Data at baseline and week 48 are median (inter-quartile range). Metabolite levels in liver samples were expressed as nanomoles per gram of liver biopsy tissue (nmol/g).The internal group comparison between baseline and 48 weeks (P*) was performed with the Wilcoxon signed-rank test. The intergroup comparison (P**) was performed with Mann-Whitney's *U* test in nonparametric parameters.

Among the hepatic amino acids, aspartic acid levels were significantly elevated in the tofogliflozin group, whereas glycine, valine, isoleucine, leucine, and lysine levels were reduced in the glimepiride group. Among the hepatic urea cycle metabolites, arginine and ornithine levels were lower in the glimepiride group (Table 1).

### 2.3 Liver histology and laboratory markers associated with the hepatic metabolite signatures

Hepatic levels of all amino acids were not associated with changes in serum liver enzymes, HbA1c levels, UCPR (urinary C-peptide–to–creatinine ratio), or BMI in either group (Supplementary Table 1).

In the glimepiride group, changes in the hepatic levels of aspartate, glycine, isoleucine, leucine, and ornithine (Δ; week 48 minus baseline) were positively associated with changes in the steatosis scores. The higher the baseline hepatic amino acid levels, the lower the hepatic steatosis scores (Table 2).

**Table 2:** The association between hepatic metabolomics and the change in histo …

	Tofogliflozin								Glimepiride							
	ΔSteatosis		ΔBallooning		ΔInflammation		ΔFibrosis		ΔSteatosis		ΔBallooning		ΔInflammation		ΔFibrosis	
	rs	P	rs	P	rs	P	rs	P	rs	P	rs	P	rs	P	rs	P
G1P at baseline	−0.185	0.508	0.051	0.858	0.003	0.992	−0.016	0.956	−0.009	0.974	−0.425	0.101	−0.076	0.781	−0.370	0.159
G6P at baseline	0.357	0.160	0.610	0.009	0.195	0.454	0.200	0.441	0.217	0.403	−0.264	0.307	−0.281	0.274	−0.238	0.358
F6P at baseline	0.415	0.110	0.583	0.018	0.181	0.501	0.227	0.398	0.197	0.449	−0.290	0.259	−0.111	0.672	−0.216	0.405
F1,6BP at baseline	0.000	1.000	0.345	0.227	−0.163	0.578	0.211	0.470	−0.427	0.127	−0.540	0.046	0.121	0.680	−0.485	0.078
acetyl-CoA at baseline	−0.401	0.196	0.102	0.753	0.172	0.593	−0.011	0.972	0.058	0.865	−0.518	0.103	−0.476	0.139	−0.641	0.034
Asp at baseline	0.061	0.815	0.105	0.688	−0.308	0.229	0.180	0.490	−0.687	0.002	−0.474	0.054	0.232	0.370	−0.351	0.167
Gly at baseline	0.052	0.844	−0.081	0.758	−0.153	0.559	0.020	0.938	−0.529	0.029	−0.316	0.216	0.215	0.406	−0.268	0.297
Val at baseline	−0.241	0.351	−0.613	0.009	−0.362	0.153	−0.089	0.733	−0.543	0.024	−0.330	0.196	0.181	0.487	−0.271	0.292
Ile at baseline	−0.276	0.284	−0.431	0.084	−0.244	0.346	0.061	0.815	−0.606	0.010	−0.303	0.237	0.323	0.206	−0.223	0.390
Leu at baseline	−0.242	0.349	−0.492	0.045	−0.291	0.257	−0.099	0.706	−0.616	0.008	−0.422	0.092	0.212	0.413	−0.329	0.197
Lys at baseline	−0.193	0.458	−0.492	0.045	−0.165	0.527	−0.151	0.564	−0.476	0.054	−0.501	0.041	0.319	0.212	−0.429	0.086
Arg at baseline	−0.410	0.102	−0.647	0.005	−0.243	0.348	0.085	0.746	−0.564	0.018	−0.211	0.417	0.405	0.107	0.086	0.743
ornithine at baseline	−0.332	0.192	−0.618	0.008	−0.435	0.081	0.155	0.552	0.205	0.431	−0.053	0.840	0.198	0.445	0.042	0.873
IMP at baseline	0.078	0.782	0.305	0.269	−0.018	0.950	0.117	0.677	−0.041	0.884	−0.454	0.089	0.175	0.532	−0.316	0.251
ATP at baseline	0.134	0.648	0.038	0.899	0.096	0.743	−0.254	0.381	0.356	0.193	−0.070	0.805	−0.353	0.197	0.235	0.398
GDP at baseline	0.473	0.075	−0.017	0.952	−0.247	0.375	−0.081	0.773	0.349	0.185	0.439	0.089	−0.064	0.814	0.390	0.136
ADP at baseline	0.195	0.454	0.469	0.058	0.091	0.727	0.157	0.548	0.275	0.286	−0.053	0.841	−0.083	0.753	0.208	0.424

ΔG1P	0.231	0.407	−0.319	0.247	−0.232	0.404	−0.049	0.863	−0.336	0.261	0.343	0.251	0.261	0.389	0.260	0.391
ΔG6P	−0.140	0.591	−0.461	0.063	−0.297	0.247	−0.292	0.256	−0.201	0.439	0.369	0.145	0.125	0.632	0.288	0.263
ΔF6P	−0.107	0.693	−0.473	0.064	−0.331	0.211	−0.272	0.308	0.425	0.130	0.583	0.029	0.070	0.813	0.233	0.423
ΔF1,6BP	0.134	0.648	−0.362	0.203	−0.015	0.960	−0.283	0.328	−0.391	0.264	0.266	0.458	0.495	0.145	0.437	0.207
Δacetyl-CoA	0.110	0.778	−0.037	0.925	0.072	0.855	0.259	0.500	−0.391	0.264	0.266	0.458	0.495	0.145	0.437	0.207
ΔAsp	0.380	0.132	0.032	0.902	−0.099	0.704	−0.048	0.856	0.610	0.009	0.343	0.178	−0.170	0.515	0.332	0.193
ΔGly	0.074	0.779	0.178	0.495	0.076	0.771	0.151	0.562	0.599	0.011	0.343	0.178	0.031	0.907	0.310	0.226
ΔVal	0.211	0.416	0.578	0.015	0.230	0.374	0.072	0.783	0.458	0.065	0.369	0.145	−0.042	0.873	0.443	0.075
ΔIle	0.221	0.395	0.594	0.012	0.281	0.275	−0.121	0.643	0.584	0.014	0.383	0.130	−0.091	0.728	0.341	0.181
ΔLeu	0.236	0.362	0.639	0.006	0.230	0.374	0.022	0.934	0.540	0.025	0.448	0.071	−0.026	0.921	0.426	0.088
ΔLys	0.159	0.543	0.659	0.004	0.253	0.327	0.140	0.591	0.642	0.005	0.356	0.161	−0.138	0.596	0.136	0.604
ΔArg	0.292	0.256	0.542	0.025	−0.052	0.843	0.121	0.645	−0.173	0.507	0.013	0.960	−0.011	0.965	0.044	0.866
Δornithine	0.240	0.354	0.647	0.005	0.260	0.313	−0.151	0.562	0.528	0.029	0.474	0.054	−0.248	0.338	0.149	0.567
ΔIMP	−0.036	0.907	−0.155	0.612	−0.079	0.797	−0.517	0.071	0.140	0.619	0.419	0.120	−0.323	0.240	0.105	0.711
ΔATP	0.196	0.541	0.016	0.962	0.020	0.952	0.004	0.991	−0.390	0.151	−0.454	0.089	0.090	0.750	−0.366	0.179
ΔGDP	−0.347	0.296	−0.026	0.941	0.326	0.328	0.273	0.417	−0.553	0.026	−0.407	0.118	0.236	0.380	−0.226	0.400
ΔADP	−0.010	0.971	−0.344	0.177	0.159	0.541	−0.323	0.206	−0.289	0.260	−0.184	0.478	−0.116	0.657	−0.349	0.170
ΔATP/ADP	−0.079	0.806	0.202	0.528	0.158	0.625	0.306	0.333	−0.347	0.206	−0.523	0.045	0.116	0.682	−0.423	0.117

The Spearman analysis performed the association. GIP, glucose 1-phosphate; G6P, glucose 6-phosphate; F6P, fructose 6-phosphate; F1,6BP, fructose 1,6-bisphosphate; Asp, aspartate; Gly, glycine; Val, valine; Ile, isoleucine; Leu, leucine; Lys, lysine; Arg, arginine; IMP, inosine monophosphate; ATP, adenosine 5′-triphosphate; GDP, guanosine 5′-diphosphate; ADP, adenosine 5′-diphosphate. Δ indicates the change from baseline to week 48 (week 48 minus baseline).

### 2.4 Changes in serum metabolite signatures

As shown in Table 3, acetylcarnitine levels were elevated during the intervention in both groups. Among the tricarboxylic acid cycle intermediates, the serum citrate and succinate levels were elevated and reduced, respectively, in the tofogliflozin group. The serum branched-chain amino acids (BCAAs; valine, leucine, and isoleucine) did not change in either group. Among the urea cycle metabolites, the urea and citrulline levels were elevated in the tofogliflozin group. Regarding butanoate metabolism, 2-hydroxyglutarate and butyrate levels were reduced in the tofogliflozin group, whereas 2-hydroxypentanoate levels were elevated in the glimepiride group.

**Table 3:** Changes in serum metabolomics parameters compared between baseline and week 48 by treatment group.

	Tofogliflozin				Glimepiride				
	N	Week 0	Week 48	P*	N	Week 0	Week 48	P*	P**
**Carbohydrate metabolism**									
lactate	20	1903.0(1759.5-2320.3)	1985.0(1666.0-2321.8)	0.794	20	2250.0(1871.5-2739.8)	2485.0(1986.3-2830.5)	0.526	0.547
**Tricarboxylic acid (TCA) cycle**									
acetylcarnitine	13	0.43(0.28-0.62)	0.55(0.49-1.22)	0.015	16	0.45(0.30-0.63)	0.60(0.32-1.28)	0.008	0.508
citrate	20	117.5(86.0-123.0)	128.5(132.3-146.5)	0.026	20	124.5(109.3-151.8)	123.5(111.8-130.8)	0.723	0.076
aconitate	20	4.9(4.2-5.9)	5.7(4.7-6.2)	0.070	20	5.6(4.4-7.1)	5.6(4.9-6.3)	0.872	0.165
succinate	20	21.0(16.3-26.3)	19.0(16.0-22.0)	0.026	20	20.5(17.5-26.8)	20.5(17.0-24.8)	0.948	0.114
fumarate	19	2.7(2.0-3.4)	2.6(2.0-3.0)	0.349	18	2.7(2.0-3.1)	2.3(2.1-3.0)	0.585	0.663
malate	20	15.0(13.3-16.8)	15.0(13.0-17.0)	0.407	20	16.5(14.3-20.5)	17.5(14.0-21.3)	0.793	0.445
**Amino acid metabolism**									
aspartate (Asp)	20	43.0(35.5-46.5)	39.5(36.0-45.0)	0.455	20	49.5(40.3-54.8)	42.0(36.3-49.8)	0.045	0.529
threonine (Thr)	20	110.0(98.5-126.8)	104.5(89.0-120.5)	0.185	20	113.0(90.8-120.0)	113.5(100.5-130.5)	0.526	0.165
asparagine (Asn)	20	14.0(11.3-20.8)	19.0(17.3-24.0)	0.001	20	16.5(11.3-19.5)	18.5(14.3-24.0)	0.006	0.134
glutamine (Gln)	18	14.0(4.2-28.8)	47.5(23.5-81.3)	<0.001	18	21.0(11.2-43.3)	29.0(19.3-111.3)	0.007	0.034
proline (Pro)	20	150.5(133.5-166.3)	148.5(134.8-166.0)	0.422	20	169.5(146.5-189.3)	176.0(157.5-196.0)	0.198	0.678
glycine (Gly)	20	189.0(170.8-221.0)	196.5(179.8-219.0)	0.304	20	188.0(159.0-247.8)	195.0(155.0-232.5)	0.896	0.512
alanine (Ala)	20	386.0(314.0-438.3)	390.0(329.5-458.8)	0.852	20	427.5(333.8-517.8)	427.0(390.8-509.3)	0.985	0.862
valine (Val)	20	257.5(234.8-269.0)	264.0(250.0-293.8)	0.126	20	271.0(147.0-303.5)	273.5(254.5-278.5)	0.401	0.659
isoleucine (Ile)	20	74.5(67.0-87.8)	76.0(63.3-93.0)	0.627	20	75.5(69.8-91.5)	79.5(70.5-87.8)	0.254	0.947
leucine (Leu)	20	141.5(133.5-160.3)	145.5(130.5-179.0)	0.103	20	158.5(142.0-175.0)	155.0(145.8-174.0)	0.550	0.429
lysine (Lys)	20	173.5(159.0-184.5)	187.5(164.8-206.3)	0.218	20	196.5(161.0-212.8)	178.0(162.3-209.3)	0.263	0.102
tyrosine (Tyr)	20	72.0(56.8-77.0)	64.5(52.0-78.8)	0.185	20	67.5(61.3-78.0)	68.5(62.3-78.8)	0.717	0.165
phenylalanine (Phe)	20	67.5(62.5-74.0)	68.5(58.5-73.8)	0.962	20	70.0(64.3-79.8)	72.0(64.5-79.8)	0.940	0.659
histidine (His)	20	48.5(43.3-52.8)	52.0(50.0-61.8)	0.006	20	56.5(50.3-60.8)	60.0(54.5-66.0)	0.061	0.301
**Urea cycle**									
arginine (Arg)	20	79.0(65.0-88.3)	78.5(62.3-86.3)	0.709	20	89.5(47.8-95.0)	78.0(62.3-94.3)	0.808	0.968
urea	20	2538.0(2291.3-2962.8)	3122.0(2596.3-3535.0)	0.001	20	2576.0(2181.0-3033.8)	2423.5(2042.5-3115.3)	0.467	0.001
ornithine	20	84.0(67.3-96.5)	90.5(69.0-122.3)	0.089	20	97.5(84.0-115.0)	89.5(71.0-110.8)	0.255	0.072
citrulline	20	24.0(19.5-28.5)	28.5(18.3-31.8)	0.029	20	20.5(15.5-29.3)	22.0(18.0-31.3)	0.150	0.862
hypoxanthine	20	20.0(14.3-24.0)	25.5(18.3-29.8)	0.073	20	21.0(15.3-27.5)	21.5(15.8-25.8)	0.711	0.192
**Butanoate metabolism**									
2-hydroxyglutarate	20	13.0(8.2-16.0)	8.3(6.4-11.0)	0.004	20	8.05(6.32-9.75)	7.00(5.85-11.00)	0.856	0.049
butyrate	18	18.0(17.0-20.0)	17.0(15.0-18.8)	0.049	18	18.0(15.8-20.0)	18.0(16.3-19.0)	0.981	0.406
2-hydroxypentanoate	18	14.0(12.0-17.5)	14.0(12.0-16.0)	0.236	20	14.5(12.0-16.8)	15.0(12.3-20.0)	0.038	0.398

Data at baseline and week 48 are median (inter-quartile range). Serum metabolite levels were expressed as concentrations in serum (μM). The internal group comparison between baseline and 48 weeks (P*) was performed with the Wilcoxon signed-rank test. The intergroup comparison (P**) was performed with Mann-Whitney's *U* test in nonparametric parameters.

### 2.5 Liver histology and laboratory markers associated with serum metabolite signatures

In the tofogliflozin group, higher baseline serum succinate levels were associated with reductions in AST, ALT, and GGT (Table 4B), and histological scores for hepatocyte ballooning and liver inflammation (Table 4A). The reduction in serum succinate levels (Δ; week 48 minus baseline) was associated with reductions in ALT and GGT levels (Table 4B). Baseline serum citrate and glutamine levels were positively associated with changes in ALT levels in the tofogliflozin group (Table 4B). In the glimepiride group, higher baseline serum succinate levels were associated with greater reductions in HbA1c levels (Table 4B), whereas decreases in serum succinate levels from baseline to week 48 were associated with improvements in hepatic fibrosis scores (Table 4A). Corresponding FDR-adjusted q values are provided in Supplementary Tables 2, 3A, and 3B.

**Table 4A:** The Association between baseline serum metabolite levels and changes in histological parameters.

	Tofogliflozin								Glimepiride							
	ΔSteatosis		ΔBallooning		ΔInflammation		ΔFibrosis		ΔSteatosis		ΔBallooning		ΔInflammation		ΔFibrosis	
	rs	P	rs	P	rs	P	rs	P	rs	P	rs	P	rs	P	rs	P
acetylcarnitine at baseline	0.522	0.150	0.825	0.006	0.299	0.434	−0.018	0.964	−0.022	0.942	−0.342	0.252	0.040	0.898	−0.134	0.662
citrate at baseline	0.082	0.731	0.134	0.572	0.226	0.339	−0.115	0.628	0.248	0.292	−0.090	0.706	−0.126	0.597	0.045	0.851
succinate at baseline	−0.208	0.378	−0.465	0.039	−0.509	0.022	0.231	0.326	0.568	0.009	0.111	0.643	−0.111	0.641	0.028	0.907
Asp at baseline	−0.251	0.286	−0.320	0.169	−0.724	<0.001	0.141	0.554	0.041	0.864	0.170	0.473	−0.094	0.693	0.170	0.474
Asn at baseline	0.453	0.045	0.395	0.084	0.116	0.628	0.332	0.153	0.074	0.756	0.171	0.471	−0.381	0.097	−0.180	0.448
Gln at baseline	0.143	0.596	0.159	0.557	0.220	0.414	0.035	0.899	−0.175	0.487	−0.258	0.301	0.434	0.072	0.061	0.809
Val at baseline	0.292	0.212	−0.032	0.895	0.086	0.718	−0.473	0.035	0.281	0.230	0.070	0.769	0.121	0.611	0.266	0.258
Ile at baseline	0.310	0.184	0.006	0.979	0.247	0.293	−0.316	0.175	0.484	0.031	0.151	0.526	0.181	0.444	0.388	0.091
Leu at baseline	0.354	0.126	−0.082	0.730	0.067	0.780	−0.493	0.027	0.181	0.444	0.191	0.421	0.039	0.871	0.343	0.138
Lys at baseline	0.350	0.131	−0.022	0.928	0.077	0.748	−0.113	0.636	0.113	0.634	0.140	0.555	−0.104	0.663	0.126	0.597
His at baseline	0.311	0.182	0.073	0.760	0.174	0.464	0.023	0.925	0.203	0.390	0.282	0.229	−0.140	0.556	−0.100	0.676
urea at baseline	0.016	0.945	−0.173	0.466	−0.230	0.329	−0.355	0.124	0.328	0.158	0.310	0.183	−0.089	0.708	0.215	0.364
citrulline at baseline	0.021	0.930	0.053	0.824	−0.243	0.302	−0.325	0.162	−0.129	0.587	0.050	0.834	−0.350	0.130	−0.266	0.256
2-hydroxyglutarate at baseline	−0.179	0.450	−0.379	0.099	−0.530	0.016	0.332	0.153	0.063	0.791	−0.100	0.674	0.027	0.911	−0.037	0.876
butyrate at baseline	0.111	0.662	−0.210	0.403	−0.255	0.307	0.042	0.869	0.021	0.933	−0.508	0.031	0.107	0.673	−0.516	0.028
2-hydroxypentanoate at baseline	0.105	0.661	−0.290	0.215	−0.067	0.777	−0.195	0.410	0.393	0.086	0.353	0.127	0.024	0.920	0.546	0.013

Δacetylcarnitine	0.173	0.656	0.596	0.090	−0.274	0.476	−0.454	0.219	−0.563	0.056	−0.209	0.514	−0.518	0.084	−0.203	0.526
Δcitrate	−0.096	0.688	−0.502	0.024	−0.169	0.477	−0.045	0.850	−0.183	0.440	0.092	0.699	0.050	0.834	−0.208	0.379
Δsuccinate	0.206	0.384	0.232	0.326	0.188	0.427	−0.010	0.966	−0.086	0.718	0.370	0.108	0.211	0.371	0.546	0.013
ΔAsp	0.281	0.231	0.371	0.107	0.010	0.967	0.015	0.950	0.120	0.615	0.284	0.225	0.191	0.421	0.412	0.071
ΔAsn	−0.274	0.242	0.044	0.853	−0.148	0.533	−0.163	0.492	−0.622	0.003	0.037	0.876	−0.432	0.057	−0.261	0.266
ΔGln	−0.195	0.470	0.301	0.258	−0.139	0.607	−0.162	0.549	−0.786	<0.001	0.057	0.827	−0.482	0.050	−0.354	0.163
ΔVal	0.090	0.705	0.059	0.804	−0.356	0.123	0.103	0.667	−0.231	0.327	−0.349	0.131	−0.100	0.674	−0.142	0.550
ΔIle	0.344	0.137	0.015	0.950	−0.268	0.253	0.100	0.674	−0.431	0.057	−0.281	0.231	−0.171	0.471	−0.200	0.397
ΔLeu	0.255	0.278	0.083	0.729	−0.199	0.400	−0.015	0.950	−0.183	0.440	−0.446	0.048	−0.210	0.373	−0.197	0.405
ΔLys	0.207	0.381	−0.227	0.336	−0.122	0.609	−0.190	0.421	−0.084	0.725	−0.210	0.374	0.050	0.834	0.238	0.312
ΔHis	−0.166	0.483	0.109	0.648	−0.100	0.674	−0.306	0.189	−0.387	0.092	−0.353	0.127	−0.171	0.471	−0.123	0.606
Δurea	−0.311	0.182	−0.195	0.409	−0.319	0.170	0.426	0.061	0.063	0.791	−0.101	0.671	0.090	0.705	−0.094	0.694
Δcitrulline	−0.138	0.561	−0.117	0.624	−0.321	0.167	−0.259	0.270	−0.473	0.035	−0.011	0.962	−0.191	0.420	0.149	0.531
Δ2-hydroxyglutarate	−0.039	0.871	0.290	0.214	0.157	0.508	−0.155	0.514	−0.058	0.808	0.473	0.035	0.130	0.584	0.355	0.125
Δbutyrate	−0.213	0.395	0.117	0.645	0.122	0.630	0.199	0.428	0.252	0.313	−0.323	0.192	0.422	0.081	0.402	0.098
Δ2-hydroxypentanoate	−0.090	0.705	0.062	0.795	−0.050	0.835	0.063	0.792	−0.064	0.787	−0.392	0.087	−0.003	0.990	−0.590	0.006

The Spearman analysis performed the association. Asp, aspartate; Asn, asparagine; Gln, glutamine; Val, valine; Ile, isoleucine; Leu, leucine; Lys, lysine; His, histidine. Δ indicates the change from baseline to week 48 (week 48 minus baseline).

**Table 4B:** The Association between baseline serum metabolite levels or metabolite changes and changes in clinical parameters.

	Tofogliflozin												Glimepiride											
	ΔAST		ΔALT		ΔGGT		ΔHbA1c		ΔBMI		ΔUCPR		ΔAST		ΔALT		ΔGGT		ΔHbA1c		ΔBMI		ΔUCPR	
	rs	P	rs	P	rs	P	rs	P	rs	P	rs	P	rs	P	rs	P	rs	P	rs	P	rs	P	rs	P
acetylcarnitine at baseline	0.500	0.170	0.450	0.225	0.126	0.747	0.460	0.213	−0.025	0.949	0.569	0.110	−0.091	0.768	0.206	0.499	0.104	0.736	−0.085	0.781	0.168	0.584	0.319	0.313
citrate at baseline	0.325	0.162	0.593	0.006	0.324	0.164	−0.007	0.976	−0.083	0.729	0.025	0.917	−0.053	0.823	−0.266	0.256	−0.029	0.905	−0.073	0.760	−0.221	0.349	−0.061	0.805
succinate at baseline	−0.586	0.007	−0.562	0.010	−0.589	0.006	−0.099	0.677	−0.273	0.243	0.156	0.510	−0.260	0.268	−0.237	0.315	0.011	0.962	0.628	0.003	0.042	0.862	0.343	0.151
Asp at baseline	−0.182	0.442	−0.155	0.514	−0.098	0.682	−0.041	0.863	−0.475	0.035	0.315	0.176	0.163	0.492	0.161	0.497	0.144	0.543	0.034	0.886	−0.037	0.877	−0.354	0.138
Asn at baseline	0.411	0.072	0.326	0.161	0.407	0.075	0.536	0.015	0.066	0.781	0.244	0.300	−0.031	0.896	−0.022	0.926	−0.291	0.213	0.030	0.901	−0.208	0.380	−0.142	0.561
Gln at baseline	0.568	0.022	0.596	0.015	0.819	<0.001	0.308	0.247	0.185	0.492	−0.133	0.625	0.095	0.707	0.203	0.420	0.179	0.478	−0.155	0.538	−0.051	0.842	−0.204	0.432
Val at baseline	0.066	0.782	−0.127	0.594	0.069	0.774	−0.189	0.426	0.392	0.087	−0.056	0.816	0.243	0.302	0.199	0.399	0.616	0.004	0.138	0.562	0.430	0.059	−0.039	0.874
Ile at baseline	0.099	0.678	−0.062	0.795	0.184	0.436	−0.240	0.308	0.466	0.038	0.128	0.592	0.267	0.255	0.144	0.546	0.589	0.006	0.081	0.734	0.184	0.438	−0.025	0.920
Leu at baseline	0.039	0.870	−0.049	0.838	0.290	0.215	−0.198	0.403	0.436	0.055	0.005	0.982	0.324	0.164	0.256	0.275	0.653	0.002	0.002	0.995	0.271	0.247	−0.175	0.474
Lys at baseline	−0.290	0.215	−0.401	0.080	−0.245	0.298	0.010	0.967	0.031	0.897	0.487	0.029	0.002	0.995	−0.080	0.739	0.332	0.152	0.188	0.428	0.080	0.738	−0.372	0.117
His at baseline	0.402	0.079	0.217	0.359	0.216	0.361	0.224	0.343	0.051	0.830	0.328	0.158	0.173	0.467	0.148	0.533	−0.031	0.895	0.054	0.821	−0.109	0.648	−0.060	0.807
urea at baseline	0.073	0.760	−0.105	0.661	0.128	0.591	−0.051	0.830	0.514	0.020	−0.273	0.244	0.271	0.248	0.313	0.179	0.289	0.216	0.242	0.303	0.209	0.376	−0.078	0.751
citrulline at baseline	−0.221	0.350	−0.118	0.621	0.074	0.755	0.095	0.689	−0.168	0.480	−0.169	0.475	−0.266	0.256	−0.087	0.717	−0.248	0.291	0.285	0.224	0.101	0.672	0.176	0.472
2-hydroxyglutarate at baseline	−0.670	0.001	−0.586	0.007	−0.496	0.026	0.033	0.892	−0.308	0.187	0.110	0.643	−0.036	0.881	−0.043	0.857	−0.014	0.955	0.015	0.950	0.103	0.665	0.137	0.577
butyrate at baseline	0.090	0.722	0.089	0.725	0.233	0.352	0.151	0.551	0.232	0.355	−0.475	0.046	0.061	0.811	−0.194	0.441	−0.088	0.729	−0.211	0.402	0.048	0.850	0.328	0.199
2-hydroxypentanoate at baseline	0.027	0.909	0.135	0.571	−0.105	0.660	−0.191	0.421	0.118	0.621	0.011	0.964	0.130	0.584	0.046	0.847	0.382	0.096	0.091	0.703	0.145	0.541	0.016	0.948

Δacetylcarnitine	−0.192	0.620	−0.276	0.472	0.301	0.431	0.000	1.000	0.317	0.406	−0.533	0.139	−0.105	0.746	−0.154	0.633	−0.032	0.922	−0.501	0.097	−0.217	0.499	0.145	0.670
Δcitrate	−0.409	0.073	−0.497	0.026	−0.343	0.139	0.005	0.982	0.065	0.787	−0.193	0.416	−0.055	0.817	0.127	0.593	−0.027	0.911	0.207	0.381	0.144	0.546	0.144	0.555
Δsuccinate	0.483	0.031	0.533	0.015	0.473	0.035	0.150	0.528	0.333	0.152	−0.348	0.133	0.225	0.340	0.090	0.705	0.456	0.043	−0.324	0.163	0.027	0.909	−0.284	0.238
ΔAsp	−0.055	0.816	−0.007	0.976	−0.060	0.803	0.079	0.740	0.404	0.077	−0.279	0.233	−0.077	0.748	−0.090	0.707	0.107	0.655	0.022	0.926	−0.073	0.759	−0.011	0.966
ΔAsn	−0.064	0.790	−0.038	0.872	0.117	0.624	−0.276	0.239	0.313	0.179	−0.237	0.315	−0.384	0.094	−0.372	0.106	−0.498	0.026	−0.376	0.102	−0.152	0.524	0.137	0.576
ΔGln	−0.112	0.680	−0.087	0.749	0.050	0.854	−0.028	0.918	0.212	0.431	−0.047	0.862	−0.216	0.405	−0.326	0.201	−0.313	0.222	−0.637	0.006	−0.205	0.430	0.093	0.733
ΔVal	−0.107	0.655	0.054	0.820	−0.037	0.876	0.238	0.313	0.045	0.850	−0.183	0.439	−0.147	0.536	−0.387	0.092	−0.323	0.165	−0.210	0.373	−0.225	0.340	0.022	0.929
ΔIle	0.115	0.629	0.087	0.715	0.052	0.828	0.482	0.032	0.309	0.185	−0.149	0.531	−0.327	0.160	−0.416	0.068	−0.419	0.066	0.006	0.980	−0.148	0.534	0.073	0.766
ΔLeu	0.170	0.473	0.189	0.424	0.170	0.474	0.250	0.289	0.284	0.224	−0.189	0.426	−0.396	0.084	−0.637	0.003	−0.366	0.113	<0.001	0.999	−0.259	0.270	0.134	0.583
ΔLys	0.168	0.479	0.272	0.246	0.325	0.163	0.323	0.165	0.382	0.096	−0.319	0.171	−0.272	0.247	−0.228	0.334	−0.298	0.202	−0.044	0.855	−0.233	0.323	0.264	0.274
ΔHis	−0.059	0.805	−0.066	0.782	−0.063	0.792	−0.285	0.223	0.261	0.266	−0.375	0.104	−0.247	0.293	−0.430	0.058	−0.379	0.100	−0.341	0.142	−0.185	0.435	0.084	0.732
Δurea	−0.161	0.497	−0.005	0.985	−0.288	0.218	0.239	0.310	−0.409	0.073	0.029	0.905	−0.379	0.099	−0.246	0.296	−0.555	0.011	0.017	0.944	−0.276	0.239	0.245	0.312
Δcitrulline	−0.273	0.244	−0.093	0.695	−0.177	0.454	−0.369	0.110	0.020	0.932	0.208	0.378	0.003	0.991	−0.229	0.332	0.074	0.757	−0.553	0.011	−0.277	0.237	−0.322	0.179
Δ2-hydroxyglutarate	0.490	0.028	0.562	0.010	0.208	0.380	−0.105	1.000	0.080	0.738	−0.106	0.656	0.014	0.952	−0.029	0.905	0.258	0.272	0.001	0.997	−0.266	0.256	−0.278	0.249
Δbutyrate	0.119	0.638	0.260	0.297	0.224	0.371	−0.083	0.742	0.003	0.990	0.499	0.035	−0.089	0.725	−0.010	0.969	0.179	0.476	0.515	0.029	0.134	0.595	−0.195	0.453
Δ2-hydroxypentanoate	0.217	0.359	0.228	0.334	0.159	0.504	0.125	0.598	0.083	0.727	−0.017	0.944	−0.106	0.657	−0.015	0.950	−0.333	0.152	0.052	0.827	0.043	0.857	0.200	0.413

The Spearman analysis performed the association. AST, aspartate transaminase; ALT, Alanine aminotransferase; GGT, Gamma-glutamyl transferase; BMI, body mass index; Asp, aspartate; Asn, asparagine; Gln, glutamine; Val, valine; Ile, isoleucine; Leu, leucine; Lys, lysine; His, histidine. Δ indicates the change from baseline to week 48 (week 48 minus baseline).

Additional analyses of volume-related clinical parameters showed that total body water assessed by impedance analyses remained unchanged in the tofogliflozin group but increased in the glimepiride group, resulting in a significant between-group difference. BUN increased significantly in the tofogliflozin group and to a greater extent than in the glimepiride group, whereas serum creatinine increased modestly in the tofogliflozin group but remained largely unchanged in the glimepiride group (Supple Table 4A). Among these parameters, changes in BUN were positively correlated with changes in serum urea in both the tofogliflozin and glimepiride groups (tofogliflozin: *r*s = 0.737, *P* < 0.001; glimepiride: *r*s = 0.872, *P* < 0.001) (Supple Table 4B), suggesting an association between circulating urea levels and dehydration-related changes.

## 3 Discussion

This is the first study to characterize the impact of antidiabetic agents on the human hepatic metabolome using paired liver biopsies. We elucidated distinct metabolic signatures between tofogliflozin and glimepiride across carbohydrate, nucleotide, and amino acid metabolism, as well as the urea cycle. By integrating serum and hepatic metabolomic data, we provide new insights into the pathophysiology of MASLD and identify potential serum biomarkers for predicting histological improvements.

T2D is characterized by impaired glucose oxidation and compensatory increases in fatty acid oxidation and gluconeogenesis. While SGLT2 inhibitors are known to modulate these pathways, our findings reveal a human-specific response. In contrast to previous reports in diabetic Akita mice at 8 weeks of age mice where dapagliflozin reversed TCA cycle perturbations [10], tofogliflozin in this study significantly elevated hepatic acetyl-CoA without altering TCA intermediates like citrate or succinate. Elevated acetyl-CoA allosterically activates pyruvate carboxylase while inhibiting pyruvate kinase [11], thereby channeling substrates toward gluconeogenesis. This aligns with our previous transcriptomic data showing the upregulation of gluconeogenic genes following treatment with tofogliflozin [7], and clinical observations of increased endogenous glucose production (EGP) under SGLT2 inhibition [12,13]. We propose that tofogliflozin enhances lipolysis and fatty acid β-oxidation, supplying the mitochondrial acetyl-CoA pool required to drive this starvation-like metabolic shift in the liver.

Intracellular citrate, produced from acetyl-CoA and oxaloacetate, can be exported to the cytosol or secreted into the circulation. In our study, tofogliflozin increased serum citrate levels, likely reflecting a steady-state balance where excess TCA cycle intermediates are either consumed by gluconeogenesis or released from the liver.

Notably, serum succinate levels were significantly reduced in the tofogliflozin group. While the exact mechanism remains to be fully elucidated, we hypothesize that SGLT2 inhibitors may facilitate succinate uptake by the liver to serve as a gluconeogenic substrate or potentially promote its consumption in thermogenic tissues like brown adipose tissue [14]. Previous experimental studies have reported increases in hepatic TCA cycle intermediates following short-term SGLT2 inhibitor treatment in mice. In contrast, hepatic TCA cycle–related metabolites remained largely unchanged after 48 weeks of tofogliflozin treatment in the present human study, suggesting metabolic adaptation during long-term therapy and maintenance of hepatic metabolic homeostasis. Furthermore, although serum succinate levels were reduced, no significant changes were observed in downstream TCA cycle intermediates, including fumarate and malate, in either serum or liver tissue. Therefore, the mechanisms underlying the reduction in circulating succinate, including its tissue origin and physiological significance, remain unclear and warrant investigation in future studies through metabolomic analyses across multiple organs.

Recently, succinate acts as an extracellular signaling molecule; succinate induces hepatic fibrogenesis by activating hepatic stellate cells via G-protein-coupled receptor 91 [15]. Indeed, serum succinate levels have been recognized as a biomarker for T2D and MASLD. The dipeptidyl peptidase 4 inhibitor gemigliptin reduced serum succinate levels and attenuated steatosis in mice fed a high fat, high cholesterol diet [16]. Baseline succinate levels reportedly predict diabetes remission after bariatric surgery [17]. Crucially, our data highlight serum succinate as a potential biomarker for MASLD severity and therapeutic response. In the tofogliflozin group, baseline succinate levels correlated with liver enzymes and histological features such as hepatocyte ballooning and a reduction in liver inflammation score. Reduction in serum succinate levels was associated with ALT and GGT levels. Also, in the glimepiride group, higher baseline serum succinate levels were associated with greater reductions in HbA1c, and decreases in serum succinate levels were associated with improvements in hepatic fibrosis scores. Since succinate is known to trigger fibrogenesis by activating hepatic stellate cells via GPR91 [15], the observed reduction in succinate levels may directly contribute to the histological improvements seen with tofogliflozin. Collectively, our findings suggest that serum succinate may represent a candidate biomarker associated with disease severity and treatment-related improvements in MASLD pathology. However, because these findings are exploratory and hypothesis-generating, further studies with larger cohorts and external validation are required to determine their clinical utility.

Our findings suggest that tofogliflozin and glimepiride generate distinct hepatic metabolic imprints, especially in hepatic amino acid handling. Tofogliflozin induced a metabolic profile resembling a fasting- or hunger-like state. It maintained BCAA levels, possibly due to a net balance between autophagy-mediated protein catabolism and consumption for the urea cycle. In addition, it increased fatty acid oxidation-mediated acetyl-CoA availability.

On the other hand, glimepiride significantly reduced hepatic BCAAs, ketogenic amino acids, and urea-cycle intermediates, likely due to insulin-mediated protein synthesis [18]. Higher baseline hepatic BCAA levels were associated with greater improvements in steatosis, and reductions in hepatic BCAA levels during treatment were also associated with improvements in steatosis scores, suggesting that increased utilization of BCAAs may contribute to reduced hepatic lipid accumulation. Collectively, these findings indicate that tofogliflozin and glimepiride improve MASLD through distinct metabolic adaptations: tofogliflozin by inducing a fasting-mimetic metabolic program and glimepiride by promoting insulin-mediated anabolic remodeling.

The urea cycle for amino acid degradation requires ammonia disposal via the urea cycle, which converts toxic ammonia to urea. Tofogliflozin elevated serum urea and urea cycle intermediates (ornithine, citrulline). This "aestivation-like" response—where urea catabolism increases to retain body fluids via osmotic mechanisms [19]—may represent a protective adaptation to the mild starvation and dehydration-like state induced by SGLT2 inhibitors. Additional analyses demonstrated that total body water increased in the glimepiride group but not in the tofogliflozin group, whereas BUN increased significantly and serum creatinine increased modestly in the tofogliflozin group. Furthermore, changes in BUN were positively correlated with changes in serum urea in both treatment groups. These findings provide additional supportfor the hypothesis of a tofogliflozin-mediated aestivation-like response in humans. It might be possible that tofogliflozin-mediated dehydration is moderated by the aestivation-like response. These metabolic adaptations likely contribute to the broader organ-protective effects of the drug class.

Because patients treated with tofogliflozin experienced significant reductions in body weight and BMI, the contribution of weight loss to the observed metabolomic alterations should be considered. However, in the primary analysis of this trial, reductions in body weight and BMI were associated with improvements in hepatic steatosis but not with changes in hepatocyte ballooning, lobular inflammation, or fibrosis [7]. Furthermore, changes in body weight were not significantly associated with the key serum or hepatic metabolites identified as treatment-related metabolic signatures in the present study. These findings suggest that the observed metabolomic alterations cannot be fully explained by weight reduction alone. However, because of the exploratory nature of this post hoc metabolomics study and the limited sample size, particularly for liver tissue analyses, multivariable regression and mediation analyses were considered underpowered and were therefore not performed. Accordingly, the relative contributions of weight loss and the direct pharmacological effects of SGLT2 inhibition could not be determined in the present study and should be clarified in future studies with larger sample sizes.

Additional limitations relate to the relatively small sample size and the large number of metabolite analyses performed. Because of the exploratory nature of the study and the limited number of participants with paired metabolomics data, non-parametric statistical methods were used rather than multivariable longitudinal models such as linear mixed-effects models. Furthermore, adjustment for multiple testing using the false discovery rate approach reduced the number of statistically significant associations. Therefore, the identified metabolite–outcome correlations should be interpreted with caution and regarded as hypothesis-generating. Future studies with larger cohorts will be required to validate the specific metabolite signatures associated with histological improvement in MASLD and to permit more comprehensive longitudinal modeling while accounting for potential confounding factors. However, the major strength of the present study is the comprehensive metabolomic analysis of paired human liver biopsy specimens and corresponding serum samples.

Finally, although serum succinate levels were associated with improvements in biochemical and histological parameters, the present study was not designed to assess biomarker performance. Accordingly, receiver operating characteristic analyses and external validation cohorts were not available. Therefore, the potential utility of serum succinate as a non-invasive biomarker should be considered preliminary. Future prospective studies are needed to determine whether serum succinate provides incremental information beyond established non-invasive markers of MASLD and to clarify its potential role in disease monitoring and treatment response assessment.

## 4 Conclusion

Tofogliflozin promotes a fasting-like metabolic signature in the human liver, characterized by elevated hepatic acetyl-CoA levels and alterations in circulating urea cycle–related metabolites consistent with a systemic adaptive response to chronic glycosuria. These shifts contrast sharply with the effects of glimepiride, which primarily promotes anabolic pathways. Furthermore, serum succinate was associated with disease severity and treatment-related improvements in MASLD, suggesting its potential as a non-invasive biomarker that warrants further validation in larger prospective studies.

## CRediT authorship contribution statement

**Yumie Takeshita:** Conceptualization, Investigation, Data curation, Formal analysis, Writing – original draft, Project administration. **Hein Ko Oo:** Formal analysis, Writing – original draft. **Yujiro Nakano:** Investigation, Data curation. **Ayano Ueno:** Investigation. **Maki Oishi:** Investigation. **Hiromasa Tsujiguchi:** Formal analysis. **Tomoyoshi Soga:** Investigation. **Taro Yamashita:** Investigation, Data curation. **Masao Honda:** Investigation, Data curation. **Toshinari Takamura:** Conceptualization, Investigation, Supervision, Writing – review & editing.

## Declaration of generative AI and AI-assisted technologies in the manuscript preparation process

The authors did not use any generative AI or AI-assisted technologies in the preparation, writing, analysis, or revision of this manuscript.

## Declaration of competing interest

TT received research grants from Kowa Company Ltd., Taisyo Pharma Co., Kyowa Kirin Co. Ltd., IQVIA Services Japan GK., Sanwa Kagaku Kenkyusho Co. Ltd., Nippon Boehringer Ingelheim Co. Ltd., Terumo Corp., Sumitomo Pharma Co. Ltd., LifeScan Japan K.K., Mitsubishi Tanabe Pharma Corp., and Abbott Japan LLC., and received lecture fees from Kowa Company Ltd., MSD K.K., Sumitomo Pharma Co. Ltd., Mitsubishi Tanabe Pharma Corp., AstraZeneca K.K., Alexion Pharma G.K., Teijin Healthcare Ltd., Kyowa Kirin Co. Ltd., Sanofi K.K., Taisyo Pharma Co., Eli Lilly Japan K.K., Novo Nordisk Pharma Ltd., Novartis Pharma., Recordati Rare Diseases Japan K.K., Nippon Boehringer Ingelheim Co. Ltd., Terumo Corp., Daiichi Sankyo Co. Ltd., Otsuka Pharmaceutical Co. Ltd., Chiesi Pharma Japan Co., and LifeScan Japan K.K., and received consulting fees from Kowa Company Ltd., Eli Lilly Japan K.K., Novo Nordisk Pharma Ltd., Teijin Healthcare Ltd., Sumitomo Pharma Co., Taisyo Pharma Co., Well-Being Management Lab., and TheracosBio, LLC. TY received research grants from JIMRO Co., Ltd., Otsuka Pharmaceutical Factory, Inc., Yakult Honsha Co., Ltd., EA Pharma Co., Ltd., Meiji Seika Pharma Co., Ltd., ASKA Pharmaceutical Co., Ltd., Astellas Pharma Inc., AstraZeneca K.K., AbbVie GK., Abbott Japan LLC., Incyte Biosciences Japan GK., Viatris Pharmaceuticals Japan GK., Eisai Co., Ltd., Otsuka Pharmaceutical Co., Ltd., Ono Pharmaceutical Co., Ltd., Gilead Sciences, K.K., Zeria Pharmaceutical Co., Ltd., Daiichi Sankyo Co., Ltd., Taisyo Pharma Co., Taiho Pharmaceutical Co., Ltd., Takeda Pharmaceutical Company Limited., Mitsubishi Tanabe Pharma Corp., Chugai Pharmaceutical Co., Ltd., Eli Lilly Japan K.K., Nippon Kayaku Co., Ltd., Novartis Pharma K.K., Miyarisan Pharmaceutical Co., Ltd., Mochida Pharmaceutical Co., Ltd., Integral Co., Ltd., and ICON Clinical Research GK. YT received research grants from Cmic Co. Ltd., ICON Clinical Research GK., Medpace Japan K.K., and IQVIA Services Japan GK.; lecture fees from Kowa Company Ltd., Sanofi K.K., Sumitomo Pharma Co. Ltd., and Novo Nordisk Pharma Ltd.; and consulting fees from Kowa Company Ltd. and Sumitomo Pharma Co. Ltd. YN received lecture fees from Kowa Company Ltd., Mitsubishi Tanabe Pharma Corp., Sumitomo Pharma Co., and Kyowa Kirin Co. Ltd.

## Data Availability

The datasets presented in this article are not readily available because of ongoing studies. Requests to access the datasets should be directed to ttakamura@med.kanazawa-u.ac.jp.

## STAR Methods

### Study design

The details of this study have been described previously [7]^,^ [20]. In brief, the present study was a 48-week, single-center (only Kanazawa University Hospital), open-label, randomized, parallel trial of tofogliflozin (fixed dose of 20 mg/day; brand name: Deberza, Kowa Company Ltd., Nagoya, Japan) and glimepiride (starting from 0.5 mg/day and titrated up to 6.0 mg/day; Sanofi-Aventis, Quebec, Canada) in adults with type 2 diabetes mellitus and biopsy-proven MASLD. All participants had a confirmed diagnosis of type 2 diabetes mellitus according to the diagnostic criteria of the American Diabetes Association. All participants were asked to fast for a minimum of 8-h fasting before each visit. In addition to studying medications, the participants continued to undergo lifestyle modifications (i.e., exercise, weight loss, and dietary adjustment) and management of various coexisting illnesses throughout the trial. The participants were asked to limit alcohol consumption to <20 g/day for women and 30 g/day for men. Previous treatment with oral antidiabetics and metabolic-related medications was continued at the same dose in all participants 12 weeks before enrollment. The participants were not allowed to receive any new prescriptions.

This study was conducted with the approval of the Certified Review Board of Kanazawa University, Ishikawa, Japan, under the guidelines stipulated in the Declaration of Helsinki. Written informed consent was obtained from all participants before their enrollment in the study. The protocol was approved by the Kanazawa University Certified Review Board, Ishikawa, Japan (2015-033, 2018-020). This trial was registered with ClinicalTrials. gov number NCT02649465 and the Japan Registry of Clinical Trials (jRCTs041180132) from UMIN 000020544.

### Participants

The eligibility criteria are discussed elsewhere [7]^,^ [20]. The inclusion criteria were as follows: 1) age ≥20 years; 2) with type 2 diabetes mellitus (HbA1c of at least 7% at 12 weeks before screening); and 3) diagnosed with “definite” MASLD and type 2 diabetes mellitus as determined by using liver biopsy specimens obtained within 12 weeks of screening.

### Randomization and Masking

Participants were randomized to the tofogliflozin or glimepiride groups in a 1:1 ratio using a computer-generated randomization sequence. Given that the current investigation was an open-label trial, the participants, investigators, and site staff were not blinded to treatment allocation. All participants received 1 h of nutritional counseling by an experienced dietitian before the 48-week treatment period. In addition, they were instructed to follow a standardized calorie diet (30 kcal/kg/day; carbohydrates, 50–60%; fat, 20–30%; and protein, 15–20%) and to engage in regular exercise (5–6 METs for 30 min daily).

### Outcomes

The outcome measure in this post hoc analysis was the change in hepatic metabolome profiles between baseline and 48 weeks of treatment. Hepatic metabolomics data were successfully generated from liver biopsy specimens of 17 participants in the tofogliflozin group and 17 participants in the glimepiride group. Serum metabolomics data were successfully generated from 20 participants in each treatment group. Metabolomic profiling could not be completed for several liver biopsy specimens because of limited tissue availability and/or technical issues during metabolomic measurements. No imputation was performed, and all analyses were conducted using the available metabolomics datasets.

## Metabolome analysis by capillary electrophoresis (CE) and mass spectrometry (MS)

### Instrumentation

Hepatic metabolome analyses were performed using liver biopsy samples obtained from participants before and after the administration of tofogliflozin or glimepiride. The liver biopsy specimens were stored in liquid nitrogen.

All CE time-of-flight MS (CE-TOFMS) experiments were performed using an Agilent CE capillary electrophoresis system (Agilent Technologies, Waldbronn, Germany), an Agilent G6230B LC/MSD TOF system (Agilent Technologies, Palo Alto, CA, USA), an Agilent1260 Infinity 2 series binary HPLC pump, a G1603A Agilent CE-MS adapter, and a G1607A Agilent CE-ESI-MS Sprayer Kit. For system control and data acquisition, we used MassHunter workstation software version B.08.00 for Agilent CE-TOFMS.

### CE-MS method

To analyze the cationic compounds, we used a fused silica capillary (50 μm i.d. × 100 cm), with 1-M formic acid as the electrolyte [21]. Approximately 5 nl of the sample solution was injected at 50 mbar for 5 s, and 30 kV of voltage was applied. The capillary temperature was maintained at 20°C and the sample tray was cooled to <5°C. Methanol/water (50% v/v) containing 0.01 μM hexakis (2,2-difluoroethoxy) phosphazene was used as a sheath liquid at 10 μL/min. ESI-TOFMS was performed in the positive ion mode, and the capillary voltage was set to 4 kV. The flow rate of heated dry nitrogen gas (heater temperature: 300°C) was maintained at 10 psi. In the TOFMS, the fragmentor, skimmer, and Oct RFV voltages were set to 75, 50, and 500 V, respectively. The automatic recalibration of each acquired spectrum was achieved using the masses of the reference standards, that is, ^13^C isotopic ion of a protonated methanol dimer (*m*/*z* 66.0631) and protonated ion of hexakis (2,2-difluoroethoxy) phosphazene (*m*/*z* 622.0290). To identify the metabolites, the relative times of migration of all peaks to the reference compounds, methionine sulfone and 3-aminopyrrolidine, were calculated, and their *m*/*z* values and relative migration times to 272 cationic metabolite standards were compared. To quantify the metabolites, the peak areas of each metabolite were corrected for the extraction efficiency of the metabolite and the sensitivity of the mass spectrometer, using methionine sulfone as an internal standard. The values were quantified from the calibration curves of 272 metabolite standards [22].

To analyze the anionic metabolites, a commercially available COSMO (+) (chemically coated with cationic polymer) capillary (50 μm i.d. × 105 cm) (Nacalai Tesque, Kyoto, Japan) was used with a 50-mM ammonium acetate solution (pH 8.5) as the electrolyte [23]. A sample solution (30 nl) was injected at 50 mbar for 30 s, and 30 kV of voltage was applied. Methanol/5-mM ammonium acetate (50% v/v) containing 0.01-μM hexakis (2,2-difluoroethoxy) phosphazene was delivered as the sheath liquid at 10 μL/min. A platinum needle was used for anionic metabolite analysis [23].

ESI-TOFMS was performed in the negative ion mode, and the capillary voltage was set to 3.5 kV. For TOFMS, the fragmentor, skimmer, and Oct RFV voltages were set to 100V, 50V, and 500V, respectively. The automatic recalibration of each acquired spectrum was performed using reference masses of the standards, that is, ^13^C isotopic ion of the deprotonated acetic acid dimer (*m/z* 120.0383) and hexakis (2,2-difluoroethoxy) phosphazene + deprotonated acetic acid (*m*/*z* 680.0355). For anion analysis, trimesate and D-camphor-10-sulfonic acid (CAS) were used as references, and CAS was used as the internal standard. To quantify the anionic metabolites, the peak areas of each metabolite corrected by CAS were compared to the calibration curves of the 237 metabolite standards. The other conditions were identical to those described in previous studies [22]^,^ [23].

The CE-TOFMS raw data were analyzed using the proprietary software MasterHands (ver. 2.17.0.10). Briefly, data processing for each experiment included data conversion, binning of data into 0.02 *m/z* slices, baseline elimination, peak picking, integration, and elimination of redundant features to yield all possible peak lists. Data matrices were generated through an alignment process based on the corrected migration times, and metabolite names were assigned to the aligned peaks by matching the *m/z* and corrected migration times of our standard library. The relative peak areas were calculated based on the ratio of the peak area to that of the internal standards, and the metabolite concentrations were calculated based on the relative peak area between the sample and the standard mixture. Metabolite levels were analyzed using the absolute quantified concentrations (serum: μM; liver: nmol/g tissue). Because the study aimed to evaluate biologically interpretable changes in metabolite abundance following treatment, no logarithmic transformation, scaling, normalization, or standardization procedures were applied prior to statistical analyses. All statistical analyses were performed using the measured metabolite concentrations.

To minimize analytical variability, all serum samples obtained at baseline and week 48 from both treatment groups were analyzed together within a single analytical run under identical experimental conditions. Internal standards were used for metabolite quantification. Because all samples were processed simultaneously rather than across multiple analytical batches or plates, batch effects and inter-plate variability were minimized, and no additional batch-effect correction or inter-plate normalization was required. Data processing, peak alignment, and quantification were performed using the standardized CE-TOFMS workflow implemented in the MasterHands software platform. Data processing, peak alignment, and quantification were performed using the standardized CE-TOFMS workflow implemented in the MasterHands software platform.

### Statistical Analysis

Statistical analyses were performed using an intention-to-treat method. We performed the Shapiro–Wilk test to evaluate the assumption of a normal distribution. Outcomes are summarized as median (interquartile range). For metabolite analyses, changes (Δ) were calculated as week 48 minus baseline values for each participant. Within-group comparisons between baseline and week 48 were performed using the Wilcoxon signed-rank test. Between-group comparisons of Δ values were performed using the Mann–Whitney *U*test because of the relatively small sample size and the non-normal distribution of metabolite concentrations.

Given the exploratory nature of this post-hoc metabolomics analysis, nominal P values were used to identify treatment-associated metabolic changes. To evaluate the potential impact of multiple testing on metabolite–outcome correlation analyses, Benjamini–Hochberg false discovery rate (FDR) correction was additionally performed as a sensitivity analysis for the correlations presented in Table 2, Table 4A, Table 4B FDR-adjusted q values are provided in the Supplementary Materials.

All analyses were performed using SPSS Statistics software (version 29.0; IBM Corp., Armonk, NY, USA).

## References

[1] EASL-EASD-EASO clinical practice guidelines on the management of metabolic dysfunction-associated steatotic liver disease (MASLD). J. Hepatol. 81, 492–542. doi: 10.1016/j.jhep.2024.04.031. 10.1016/j.jhep.2024.04.031 38851997

[2] Hamaguchi E., Takamura T., Sakurai M. (2010). Histological course of nonalcoholic fatty liver disease in Japanese patients: tight glycemic control, rather than weight reduction, ameliorates liver fibrosis. Diabetes Care.

[3] Sako S., Takeshita Y., Takayama H. (2023). Trajectories of liver fibrosis and gene expression profiles in nonalcoholic fatty liver disease associated with diabetes. Diabetes.

[4] Younossi Z.M., Mangla K.K., Berentzen T.L. (2024). Liver histology is associated with long-term clinical outcomes in patients with metabolic dysfunction-associated steatohepatitis. Hepatol. Commun..

[5] Miao L., Targher G., Byrne C.D. (2024). Current status and future trends of the global burden of MASLD. Trends Endocrinol. Metabol..

[6] Ng C.H., Lim W.H., Lim G.E.H. (2023). Mortality outcomes by fibrosis stage in Nonalcoholic Fatty liver disease: a systematic review and meta-analysis. Clin. Gastroenterol. Hepatol..

[7] Takeshita Y., Honda M., Harada K. (2022). Comparison of tofogliflozin and glimepiride effects on nonalcoholic fatty liver disease in participants with type 2 diabetes: a randomized, 48-week, open-label, active-controlled trial. Diabetes Care.

[8] Ong Lopez A.M.C., Pajimna J.A.T. (2024). Efficacy of sodium glucose cotransporter 2 inhibitors on hepatic fibrosis and steatosis in non-alcoholic fatty liver disease: an updated systematic review and meta-analysis. Sci. Rep..

[9] Furuya F., Fujita Y., Matsuo N. (2022). Liver autophagy-induced valine and leucine in plasma reflect the metabolic effect of sodium glucose co-transporter 2 inhibitor dapagliflozin. EBioMedicine.

[10] Kogot-Levin A., Riahi Y., Abramovich I. (2023). Mapping the metabolic reprogramming induced by sodium-glucose cotransporter 2 inhibition. JCI Insight.

[11] Perry R.J., Camporez J.P.G., Kursawe R. (2015). Hepatic acetyl-CoA links adipose tissue inflammation to hepatic insulin resistance and type 2 diabetes. Cell.

[12] Vallon V. (2024). State-of-the-Art-Review: mechanisms of action of SGLT2 inhibitors and clinical implications. Am. J. Hypertens..

[13] Merovci A., Solis-Herrera C., Daniele G. (2014). Dapagliflozin improves muscle insulin sensitivity but enhances endogenous glucose production. J. Clin. Investig..

[14] Mills E.L., Pierce K.A., Jedrychowski M.P. (2018). Accumulation of succinate controls activation of adipose tissue thermogenesis. Nature.

[15] Park S.Y., Le C.T., Sung K.Y., Choi D.H., Cho E.H. (2018). Succinate induces hepatic fibrogenesis by promoting activation, proliferation, and migration, and inhibiting apoptosis of hepatic stellate cells. Biochem. Biophys. Res. Commun..

[16] Nguyen G., Park S.Y., Do D.V., Choi D.H., Cho E.H. (2022). Gemigliptin alleviates succinate-induced hepatic stellate cell activation by ameliorating mitochondrial dysfunction. Endocrinol. Metab..

[17] Ceperuelo-Mallafré V., Llauradó G., Keiran N. (2019). Preoperative circulating succinate levels as a biomarker for diabetes remission after bariatric surgery. Diabetes Care.

[18] Tessari P. (2024). Stepwise discovery of insulin effects on amino acid and protein metabolism. Nutrients.

[19] Marton A., Kaneko T., Kovalik J.P. (2021). Organ protection by SGLT2 inhibitors: role of metabolic energy and water conservation. Nat. Rev. Nephrol..

[20] Takeshita Y., Kanamori T., Tanaka T. (2020). Study protocol for pleiotropic effects and safety of sodium-glucose cotransporter 2 inhibitor versus sulfonylurea in patients with type 2 diabetes and nonalcoholic fatty liver disease. Diabetes Ther.

[21] Soga T., Ohashi Y., Ueno Y., Naraoka H., Tomita M., Nishioka T. (2003). Quantitative metabolome analysis using capillary electrophoresis mass spectrometry. J. Proteome Res..

[22] Soga T., Baran R., Suematsu M. (2006). Differential metabolomics reveals ophthalmic acid as an oxidative stress biomarker indicating hepatic glutathione consumption. J. Biol. Chem..

[23] Soga T., Igarashi K., Ito C., Mizobuchi K., Zimmermann H.P., Tomita M. (2009). Metabolomic profiling of anionic metabolites by capillary electrophoresis mass spectrometry. Anal. Chem..

