# Supplementary material for: Distinct metabolomic signatures of SGLT2 inhibitor vs. sulfonylurea in a paired human liver biopsy study
Source: Biochem Biophys Rep. 2026 Sep 14;48:102784. doi: 10.1016/j.bbrep.2026.102784 (PMC13594538; doi:10.1016/j.bbrep.2026.102784)
Supplement: Supplementary file 1 [file mmc1.docx]

Additional Appendix

**Suppl. Table 1.** **The association between hepatic metabolomics and the change of laboratory data in both groups**

The Spearman analysis performed the association. AST, aspartate transaminase; ALT, Alanine aminotransferase; GGT, Gamma-glutamyl transferase; BMI, body mass index; GIP, glucose 1-phosphate; G6P, glucose 6-phosphate; F6P, fructose 6-phosphate; F1,6BP, fructose 1,6-bisphosphate; Asp, aspartate; Thr, threonine; Ser, serine; Asn, asparagine; Gln, glutamine; Pro, proline; Gly, glycine; Ala, alanine; Val, valine; Ile, isoleucine; Leu, leucine; Lys, lysine; Tyr, tyrosine; Phe, phenylalanine; His, histidine; Arg, arginine; IMP, inosine monophosphate; ATP, adenosine 5'-triphosphate; AMP, adenosine monophosphate; GDP, guanosine 5'-diphosphate; ADP, adenosine 5'-diphosphate; UDP, uridine 5'-diphosphate; UTP, uridine 5'-triphosphate

**Suppl. Table 2.** **The association between hepatic metabolomics and the change in histology in both groups**

The Spearman analysis performed the association. GIP, glucose 1-phosphate; G6P, glucose 6-phosphate; F6P, fructose 6-phosphate; F1,6BP, fructose 1,6-bisphosphate; Asp, aspartate; Gly, glycine; Val, valine; Ile, isoleucine; Leu, leucine; Lys, lysine; Arg, arginine; IMP, inosine monophosphate; ATP, adenosine 5'-triphosphate; GDP, guanosine 5'-diphosphate; ADP, adenosine 5'-diphosphate. P values are nominal P values. q values were calculated using the Benjamini–Hochberg false discovery rate (FDR) correction.

**Suppl. Table 3A. The Association between baseline serum metabolite levels and changes in histological parameters**

The Spearman analysis performed the association. Asp, aspartate; Asn, asparagine; Gln, glutamine; Val, valine; Ile, isoleucine; Leu, leucine; Lys, lysine; His, histidine. Δ indicates the change from baseline to week 48 (week 48 minus baseline). P values are nominal P values. q values were calculated using the Benjamini–Hochberg false discovery rate (FDR) correction.

**Suppl. Table 3B. The Association between baseline serum metabolite levels or metabolite changes and changes in clinical parameters**

The Spearman analysis performed the association. Asp, aspartate; Asn, asparagine; Gln, glutamine; Val, valine; Ile, isoleucine; Leu, leucine; Lys, lysine; His, histidine; AST, aspartate transaminase; ALT, Alanine aminotransferase; GGT, Gamma-glutamyl transferase; BMI, body mass index. P values are nominal P values. q values were calculated using the Benjamini–Hochberg false discovery rate (FDR) correction.

**Suppl. Table 4A. Changes in laboratory data related to the urea cycle compared between baseline and week 48 by treatment group**

Data at baseline and week 48 are median (inter-quartile range). The internal group comparison between baseline and 48 weeks (P*) was performed with the Wilcoxon signed-rank test. The intergroup comparison (P**) was performed with Mann-Whitney’s U test in nonparametric parameters.

**Suppl. Table 4B. The Association between the changes in the urea cycle metabolites and changes in clinical parameters**

The Spearman analysis performed the association. Δ indicates the change from baseline to week 48 (week 48 minus baseline). P values are nominal P values. q values were calculated using the Benjamini–Hochberg false discovery rate (FDR) correction.
